# Population Impact and Effectiveness of Monovalent Rotavirus Vaccination in Urban Malawian Children 3 Years After Vaccine Introduction: Ecological and Case-Control Analyses

**DOI:** 10.1093/cid/civ1183

**Published:** 2016-04-07

**Authors:** Naor Bar-Zeev, Khuzwayo C. Jere, Aisleen Bennett, Louisa Pollock, Jacqueline E. Tate, Osamu Nakagomi, Miren Iturriza-Gomara, Anthony Costello, Charles Mwansambo, Umesh D. Parashar, Robert S. Heyderman, Neil French, Nigel A. Cunliffe

**Affiliations:** 1 Malawi-Liverpool-Wellcome Trust Clinical Research Programme, College of Medicine, University of Malawi, Blantyre; 2 Institute of Infection and Global Health, University of Liverpool, United Kingdom; 3 Epidemiology Branch, Division of Viral Diseases, National Center for Immunization and Respiratory Diseases, Centers for Disease Control and Prevention, Atlanta, Georgia; 4 Graduate School of Biomedical Sciences, Nagasaki University, Japan; 5 Institute of Global Health, University College London, United Kingdom; 6 Ministry of Health, Lilongwe, Malawi; 7 Liverpool School of Tropical Medicine; 8 Division of Infection and Immunity, University College London, United Kingdom

**Keywords:** rotavirus vaccine, population impact, vaccine effectiveness, developing countries, case-control

## Abstract

***Background.*** Rotavirus vaccines have been introduced in many low-income African countries including Malawi in 2012. Despite early evidence of vaccine impact, determining persistence of protection beyond infancy, the utility of the vaccine against specific rotavirus genotypes, and effectiveness in vulnerable subgroups is important.

***Methods.*** We compared rotavirus prevalence in diarrheal stool and hospitalization incidence before and following rotavirus vaccine introduction in Malawi. Using case-control analysis, we derived vaccine effectiveness (VE) in the second year of life and for human immunodeficiency virus (HIV)–exposed and stunted children.

***Results.*** Rotavirus prevalence declined concurrent with increasing vaccine coverage, and in 2015 was 24% compared with prevaccine mean baseline in 1997–2011 of 32%. Since vaccine introduction, population rotavirus hospitalization incidence declined in infants by 54.2% (95% confidence interval [CI], 32.8–68.8), but did not fall in older children. Comparing 241 rotavirus cases with 692 test-negative controls, VE was 70.6% (95% CI, 33.6%–87.0%) and 31.7% (95% CI, −140.6% to 80.6%) in the first and second year of life, respectively, whereas mean age of rotavirus cases increased from 9.3 to 11.8 months. Despite higher VE against G1P[8] than against other genotypes, no resurgence of nonvaccine genotypes has occurred. VE did not differ significantly by nutritional status (78.1% [95% CI, 5.6%–94.9%] in 257 well-nourished and 27.8% [95% CI, −99.5% to 73.9%] in 205 stunted children; *P* = .12), or by HIV exposure (60.5% [95% CI, 13.3%–82.0%] in 745 HIV-unexposed and 42.2% [95% CI, −106.9% to 83.8%] in 174 exposed children; *P* = .91).

***Conclusions.*** Rotavirus vaccination in Malawi has resulted in reductions in disease burden in infants <12 months, but not in older children. Despite differences in genotype-specific VE, no genotype has emerged to suggest vaccine escape. VE was not demonstrably affected by HIV exposure or stunting.

Following randomized trial evidence of rotavirus vaccine efficacy in low-income settings [1] that led the World Health Organization (WHO) to recommend global implementation [2], as of August 2015, 35 low-income African countries have introduced rotavirus vaccination into their Expanded Programme on Immunization schedules with support from Gavi, the Vaccine Alliance [3]. Monovalent rotavirus vaccine (RV1) effectiveness (VE) and cost-effectiveness have been demonstrated following vaccine rollout in low-income, high-burden settings [4–6]. In Malawi, one of the first African countries to introduce rotavirus vaccine into its national immunization program in 2012, RV1 reduced population rotavirus hospitalization burden by 43% (95% confidence interval [CI], 18%–61%) with an effectiveness compared to test-negative controls of 64% (95% CI, 24%–83%) against severe rotavirus gastroenteritis [5].

Despite early evidence of rotavirus vaccine impact in low-income settings, it remains important to determine VE against additional endpoints of public health significance, particularly with an accelerated immunization schedule at 6 and 10 weeks that was not examined in clinical trials. Demonstrating persistence of protection beyond infancy is important as previous case-control studies in South America and a randomized trial in Malawi, respectively, found lower effectiveness and efficacy in second year of life, suggesting the possibility of waning immunity [7–9]. Likewise, the utility of the WHO-scheduled vaccine in specific high-risk subgroups, such as malnourished or human immunodeficiency virus (HIV)–exposed children, has not been fully established. Poor nutrition is associated with gastrointestinal morbidity [10], and HIV-exposed children (those born to an HIV-infected mother) face persistent immunological defects and a higher disease burden, even if they are uninfected with HIV [11, 12]. In a randomized trial in South Africa, RV1 produced satisfactory immune responses in HIV-infected infants [13, 14], and subsequent case-control studies with a second dose at 14 weeks showed comparable VE among HIV-exposed but uninfected and HIV-unexposed children [4]. The effectiveness among these risk groups of the WHO globally recommended 6- and 10-week schedule has not been investigated.

A wide diversity of rotavirus strains has been reported in the past 2 decades in Malawi, with emergence of G8 genotypes in the 1990s [15], G12 in the mid-2000s [16] and G2 just prior to vaccine introduction in 2012 [5]. Additionally, despite trial evidence of heterotypic (cross-serotype) protection provided by the monovalent G1P[8] vaccine [17], confirming genotype-specific VE and the absence of vaccine escapes is important [18].

Utilizing an existing surveillance platform in Blantyre, Malawi [19], to extend our early observations [5], we sought to address questions of waning effectiveness with age, of effectiveness in select high-risk populations, and of effectiveness against a variety of circulating strains. We have analyzed prevaccine, sentinel hospital-based surveillance dating back to 1997 [16] and undertaken postvaccine case-control studies [5].

## METHODS

### Baseline Surveillance

From 1 January 1997 to 31 July 2009, we conducted surveillance for diarrheal disease at the Queen Elizabeth Central Hospital (QECH) in Blantyre, Malawi [16, 20]. QECH is a government-funded teaching hospital for the southern region of Malawi, and provides free healthcare to a population of about 1.3 million persons. It is the referral facility for a network of 23 government primary health centers. We recruited children aged <5 years presenting with acute diarrhea to QECH. Study nurses actively recruited children in the Accident and Emergency Department Monday through Friday, and selectively sought to capture all admitted children and those with short-stay (approximately 4 hours) for observed oral rehydration.

### Enhanced Surveillance

From January 2012, our surveillance activities at this site were enhanced in light of impending introduction of rotavirus vaccine and included additional inpatient pediatric wards (nursery, malnutrition, main ward, and special care ward) with Monday through Saturday surveillance (Sunday admissions were usually recruited on Monday morning). Since January 2012 we obtained demographic, clinical, and anthropometric data through parental interview and review of medical notes and physical examination. Severity was measured using the Ruuska–Vesikari scale [21], and stunting was defined as length-for-age *z* score < −2. We obtained rotavirus vaccination status from government-issued patient-held vaccine record (the “health passport”) and excluded from analysis those with a missing record. These surveillance platforms and case ascertainment methods have been described in detail previously [5, 19].

### Laboratory Methods

During both surveillance periods, we collected stool for rotavirus testing by enzyme immunoassay (EIA) (Rotaclone, Meridian Bioscience, Cincinnati, Ohio). EIA-positive stools underwent VP7 (G) and VP4 (P) genotyping using qualitative, heminested multiplex reverse transcription–polymerase chain reaction (PCR) as previously described [22]. We screened all EIA-positive stools collected from vaccinated children for vaccine strain shedding using a RV1 NSP2-specific quantitative PCR assay [23]. HIV status of the mother was obtained from her health passport, or was determined from the child finger-prick blood samples using 2 sequential antibody rapid tests (Determine, Abbott Laboratories, Germany; Uni-Gold, Trinity Biotech, Ireland) or by DNA PCR in infants aged <12 months according to national guidelines [24]. A child was considered HIV exposed but uninfected if the mother was documented as HIV infected or tested positive on sequential rapid test but her child had negative rapid test alone or a negative DNA result regardless of rapid test result. Children whose mother's status was unknown and who themselves had a negative rapid test were considered unexposed.

### Analysis

Because our surveillance in the year before and since vaccine introduction was enhanced, we cannot directly compare population-based incidence rates for the 2 surveillance periods (from 1 January 1997 to 31 July 2009 and from January 2012 onward, respectively). Thus, we relied on the comparison of rotavirus prevalence in diarrheal stools across these periods, using the Royston χ^2^ test for trend to test the null hypothesis of no change in prevalence over time [25]. We report Wilson confidence bounds around binomial proportions [26]. We also present genotyping data from our historical archive, and compare historical baseline genotype-specific prevalence in diarrheal stool with current prevalence in the post–rotavirus vaccine era.

For the second surveillance period, we calculated population incidence of hospitalized rotavirus and of genotype-specific rotavirus in infants <12 months old and in children aged 1–4 completed years, as the number of cases observed divided by 100 000 age-specific Blantyre population derived from midyear population projections from the 2008 population census [27]. Projections were derived through linear extension of annual increase in age-specific population in the intercensal period going back to 1998. We then calculated the ratio of the incidence rate for the period 1 January to 30 June 2012 before vaccine was introduced to the rate in equivalent calendar periods for 2013, 2014, and 2015. We report vaccine impact as 1 minus this incidence rate ratio [28]. Because there was no catch-up campaign when rotavirus vaccine was introduced to Malawi, for children aged >1 year we compare the rates in 2014 and 2015 against the mean rate for 2012 and 2013. This is because 2013 was effectively a prevaccine year for this group. For each year we report the impact compared to baseline together with the calculated VE derived for these same years. VE is derived from logistic regression as 1 minus the adjusted odds ratio (OR) of receiving 2 doses of rotavirus vaccine in EIA-confirmed rotavirus cases compared with diarrheal EIA-negative controls. We adjusted the OR for year and month of presentation and for age. In addition, our study protocol defined as secondary endpoints the evaluation of VE by year of age, by genotype, in HIV-exposed children and in malnourished children. For year of age and genotype, we derived VE using the defined subgroup as cases and comparing rotavirus-negative controls. In the case of HIV and malnutrition, we also conducted stratified analysis comparing VE among children with the condition of interest against the VE in children without the condition, and tested the null hypothesis of homogeneity of the VE across strata using the Cochran-Mantel-Haenszel test [29]. We first ensured no significant interaction between the strata of interest and vaccine status (results not shown). All VE estimates include data from the date of introduction 29 October 2012 to 30 June 2015. Analysis was conducted using Stata 12.1 (StataCorp, College Station, Texas). The endpoints reported in this paper were protocol predefined but represent unpowered secondary analyses.

### Ethics

Ethical approval was provided by the National Health Sciences Research Committee, Lilongwe, Malawi (867), and by the Research Ethics Committee of the University of Liverpool, United Kingdom (000490). Written consent was obtained from the parents or guardians of participating children.

## RESULTS

### Vaccine Coverage Since Introduction

Among vaccine age-eligible infants <12 months presenting with rotavirus EIA-negative diarrhea, vaccine coverage with 2 doses of RV1 was 74.6% in 2013, 92.4% in 2014, and 95.1% in 2015. Among rotavirus-negative children >1 year of age, the coverage rates were, respectively 18.4%, 70.1%, and 87.3%.

### Long-term Prevalence of Rotavirus and Specific Genotypes Over Time

**Figure 1.**
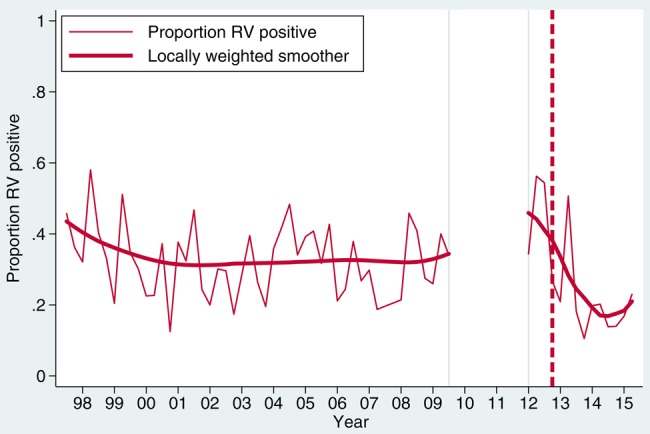
Rotavirus (RV) prevalence in diarrheal stools at Queen Elizabeth Central Hospital, Blantyre, Malawi, 1997–2015.

**Figure 2.**
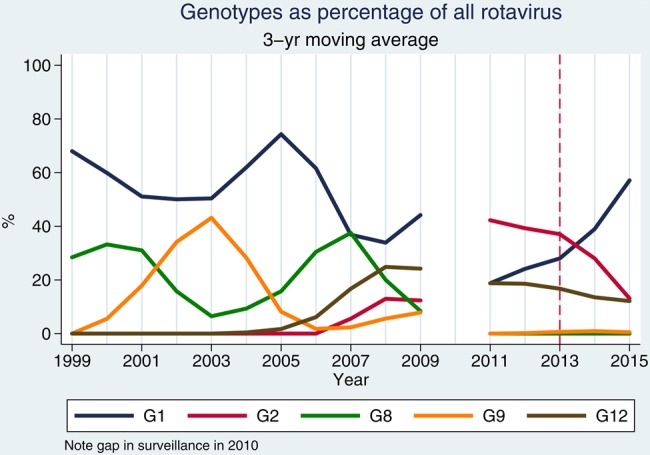
Three-year moving average of genotype-specific prevalence in rotavirus enzyme immunoassay–positive stools, 1999–2009 and 2011–2015.

**Figure 3.**
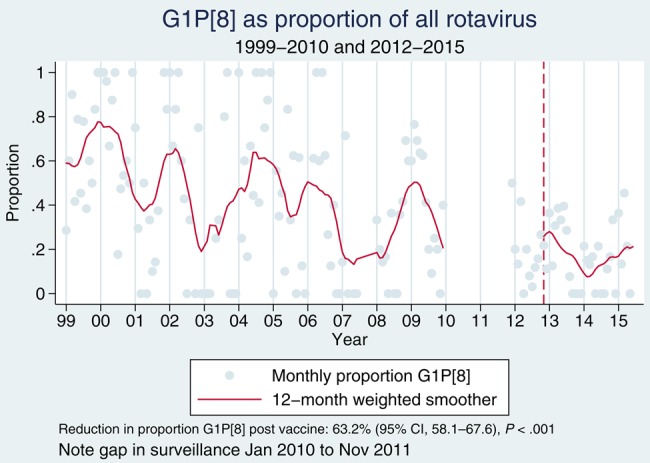
G1P[8] prevalence among rotavirus enzyme immunoassay–positive diarrheic stools at Queen Elizabeth Central Hospital, Blantyre, Malawi, 1 January 1999–31 December 2009 and January 2012–June 2015. Abbreviation: CI, confidence interval.

Between 1 July 1997 and 30 June 2015, we recruited 5875 children with diarrhea. A comparison of postvaccine rotavirus prevalence among children with diarrhea aged <5 years against our historical archive shows lower prevalence than in the prior decade of surveillance (Figure 1). Annual prevalence in the prevaccine years 1997–2009 was 32.4% (Wilson 95% CI, 31.1%–33.8%), whereas in the postvaccine years 2013–2015 it was 29.3% (Wilson 95% CI, 27.0%–31.7%) (*P* = .029). In the period January–June of 2012–2015, rotavirus prevalence in stool was 44.1%, 41.7%, 29.1%, and 24.3%, respectively. Genotype-specific prevalence in diarrheal rotavirus-EIA positive stool varied from year to year and no long-term trend is apparent (Figure 2). However for G1P[8], prevalence is lower since vaccine introduction than at any time during historical surveillance at our site (Figure 3). In the calendar periods 1 January–30 June of 2014 and 2015 combined, G1P[8] has had a nonsignificant decline of 54.0% (95% CI, −13.4% to 81.3%; *P* = .109) compared with prevaccine baseline of 1 January to 30 June 2012. On specific testing, none of the G1 rotaviruses was vaccine virus (data not shown). A transient increase was observed for G2P[4] from January 2012 to April 2014, but overall for the same period of 1 January–30 June of 2014 and 2015 compared with preintroduction levels, there was a nonsignificant increased incidence of G2P[4] of 10.5% (95% CI, −61.1% to 213.5%; *P* = .88).

### Rotavirus Hospitalization Incidence in Infants <12 Months, 2012 to 2015

**Figure 4.**
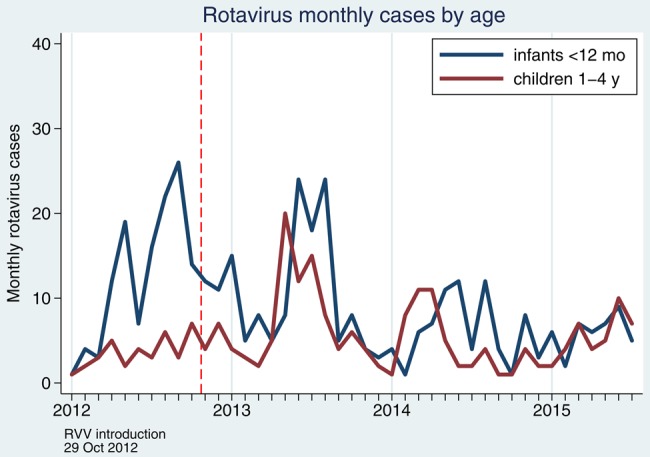
Monthly cases of rotavirus at Queen Elizabeth Central Hospital, Blantyre, Malawi, 1 January 2012–30 June 2015. Abbreviation: RVV, rotavirus vaccine.

Population incidence of rotavirus hospitalization and rotavirus prevalence in diarrheal stool during the enhanced surveillance period 2012–2015 are presented in Table 1. There was a significant reduction in population incidence of rotavirus hospitalization in infants over time. A before–after comparison of January–June 2012 (prevaccine) with the mean incidence for January–June of the years 2013–2015 shows a reduction in infants of 48.2% (95% CI, 36.5%–57.7%; *P* < .0001). A year-by-year comparison for each January–June periods compared to 2012 in infants showed no reduction in 2013, a reduction of 43.2% (95% CI, 18.0%–60.7%; *P* = .0026) in 2014, and of 54.2% (95% CI, 32.8%–68.8%; *P* < .0001) in 2015 (Table 1 and Figure 4).

**Table 1.** Rotavirus Hospitalization Incidence and Rotavirus Prevalence in Diarrheic Stool, 2012–2015

Year	Infants <1 y				Children 1–4 y			
	Incidence^a^	Rotavirus EIA		Total	Incidence^a^	Rotavirus EIA		Total
		No. Positive	No. Negative			No. Positive	No. Negative	
2012	268.7	79 (49%)	82 (51%)	161 (100%)	32.8	19 (28%)	48 (71%)	67 (100%)
2013	284.2	87 (40%)	132 (60%)	219 (100%)	114.6	57 (46%)	68 (54%)	125 (100%)
2014	152.5	52 (31%)	115 (69%)	167 (100%)	70.2	39 (27%)	107 (73%)	107 (100%)
2015	123.1	42 (23%)	144 (77%)	186 (100%)	60.3	37 (24%)	115 (76%)	152 (100%)
	Total	260	473	733	Total	152	338	490
		Pearson χ^2^ = 29.6, *P* < .001 Royston χ^2^ for trend = 29.6, *P* < .0001				Pearson χ^2^ = 17.1, *P* = .001 Royston χ^2^ for trend = 5.5, *P* = .019		

Abbreviation: EIA, enzyme immunoassay.

^a^ Incidence from January to June per 100 000 age-adjusted Blantyre population.

### Rotavirus Hospitalization Incidence in Children Aged 1–4 Years, 2012–2015

RV1 was introduced in Malawi without any catch-up campaign, so children aged 1 year and older were ineligible to receive vaccine until October 2013. In comparison to January–June 2013, the same calendar months in 2014 saw a decline of 38.7% (95% CI, 6.3%–59.9%; *P* = .024) and in 2015 of 47.4% (95% CI, 18.4%–66.1%; *P* = .004). But when comparing the mean incidence for January–June of the years 2013–2015 against a baseline of January–June 2012 (prevaccine), an increase in population incidence of 38.5% (95% CI, −1.9% to 95.4%; *P* = .06) was found. Given year-on-year variability in incidence in this group, discerning any trend is difficult (Table 1 and Figure 4).

### Rotavirus Age Distribution

**Figure 5.**
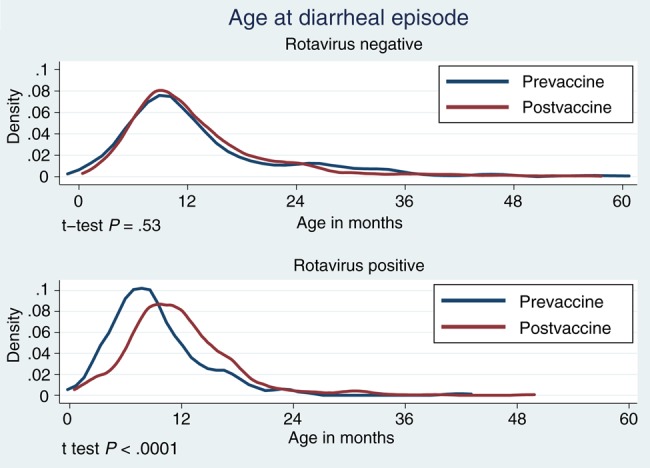
Age at diarrheal episode by rotavirus status before (1 January 2012–28 October 2012) and after (29 October 2012–30 June 2015) monovalent rotavirus vaccine introduction.

Since vaccine introduction, rotavirus cases have occurred at an older age; the mean age in months was 9.3 (standard deviation [SD], 5.2) preintroduction and is now 11.8 (SD, 5.8) months (*P* < .001). In 2015, children >1 year of age constituted 42 (46.7%) of 90 rotavirus cases. No age shift occurred in nonrotavirus diarrhea cases (mean age in months, 13.5 [SD, 9.5] preintroduction and 13.1 [SD, 8.3] postintroduction; *P* = .53; Figure 5).

### Rotavirus Vaccine Effectiveness

Crude VE overall since vaccine introduction was 60.4% (95% CI, 25.4%–79.0%; *P* = .004), whereas adjusting for age, year, and month of admission gave VE of 58.3% (95% CI, 20.2%–78.2%).

VE estimates by age group, by HIV status, by nutrition status, by disease severity, and by genotype are shown in Table 2. Notably, the point estimate of VE was markedly lower in children in the second year of life than in infants, though fewer rotavirus cases in this age group result in wide confidence bounds. Although the number of HIV-exposed but uninfected children was not high, VE was of comparable magnitude to that in unexposed children, and there was no evidence that VE differs by HIV exposure status. In well-nourished children, the point estimate of VE was substantially higher than in stunted children, but the confidence bounds were wide and this difference was not statistically significant. There was no obvious relationship between VE and disease severity measured by Ruuska-Vesikari score. Despite a comparable number of G2 and G12 genotypes to G1 genotypes, the point estimate of VE was lower and not significant against the former genotypes, and was significant and higher against the G1 genotype (Table 2). Correspondingly, VE was comparable for P[6] and P[8], but against P[4] VE was lower and nonsignificant (Table 2).

**Table 2.** Adjusted^a^ Vaccine Effectiveness in Children by Subgroup

Subgroup	Cases/Controls, No.	2-Dose VE, % (95% CI)	*P* Value
All	241/692	58.3 (20.2–78.2)	.008
Age <12 mo	167/467	70.6 (33.6–87.0)	.003
Age 12–23 mo	71/201	31.7 (−140.6 to 80.6)	.552
Age 12–31 mo^b^	73/225	28.8 (−147.5 to 79.5)	.594
HIV unexposed	191/554	60.5 (13.3–82.0)	.021
HIV exposed and uninfected^c^	48/126	42.2 (−106.9 to 83.8)	.400
CMH test			.912
Well nourished^d^	74/183	78.1 (5.6–94.9)	.042
Stunted^e^	53/152	27.8 (−99.5 to 73.9)	.530
CMH test			.115
Vesikari score ≤10^f^	42/187	66.3 (−5.0 to 89.2)	.061
Vesikari score >10	149/368	59.7 (9.3–82.1)	.028
Vesikari score >15	49/116	65.2 (−16.5 to 89.6)	.087
G1P[8]^g^	36/692	82.1 (44.6–94.2)	.003
G2P[4]	43/692	34.9 (−135.0 to 82.0)	.512
G1 (any P type)	98/692	70.7 (20.1–89.3)	.016
G2 (any P type)	61/692	45.9 (−47.0 to 80.1)	.228
G12 (any P type)	38/692	51.0 (−88.5 to 87.3)	.299
P[4] (any G type)	58/692	32.8 (−109.1 to 78.4)	.493
P[6] (any G type)	72/692	68.1 (14.9–88.1)	.022
P[8] (any G type)	50/692	71.0 (20.6–89.4)	.016
Entirely heterotypic: any non-G1, non-P[8]	112/692	46.6 (−21.7 to 76.6)	.136

Abbreviations: CI, confidence interval; CMH, Cochran–Mantel–Haenszel test of homogeneity across strata [29]; HIV, human immunodeficiency virus; VE, vaccine effectiveness.

^a^ All analyses adjusted for age, year, and month of admission.

^b^ Oldest vaccine age-eligible case was 31 months old.

^c^ Analysis restricted to exposed uninfected comparing rotavirus enzyme immunoassay (EIA) positive to negative. Two HIV-infected children were not included in analysis.

^d^ Weight-for-age, length-for-age, and weight-for-length *z* score all > −2 and mid-upper arm circumference > 11 cm.

^e^ Analysis restricted to stunted (length-for-age *z* score ≤ −2) comparing rotavirus EIA positive to negative.

^f^ Analysis restricted to stated Vesikari score range comparing rotavirus EIA positive to negative.

^g^ All specific genotypes compared with EIA negative.

## DISCUSSION

In the current post–rotavirus vaccine era in Malawi, rotavirus prevalence rates are the lowest since surveillance began almost 18 years ago [16]. Each year since vaccine introduction and concurrent with increasing vaccine coverage, we have observed successive reductions in population incidence of rotavirus hospitalization. We found sustained VE of 58.3% (95% CI, 20.2%–78.2%), which is comparable to VE estimates reported in a prior clinical trial in Malawi [1]. However, consistent with prior studies [7–9] we also observed lower VE in children aged 1–2 years and no evident declines in incidence in children >1 year old. Although it is plausible that in the presence of herd protection, unvaccinated children are less exposed to disease, thereby lowering apparent VE, modeling has shown the impact of such epidemiological phenomena to be marginal [30]. In our population with high vaccine coverage, we have observed an increase in the mean age of rotavirus cases, but not of rotavirus-negative diarrhea cases. The absolute burden of disease in the older age group has not increased, however. This is consistent with a reduction in the burden of hospital-attended disease disproportionately affecting those who have most recently had the vaccine and a time-dependent decay in VE. This finding suggests waning immunity and will require continued monitoring. If herd protection is not achieved with this vaccine, waning immunity is likely to manifest as resurgence in disease in older groups, and this should be detectable using consistent surveillance methods.

While RV1 is known to provide heterotypic protection [17], we found higher point estimates of VE against the G1 genotype, and highest of all against fully homotypic G1P[8] genotypes, and lowest for totally heterotypic strains. We have previously reported on the dominance of G2 in the season following vaccine introduction in Malawi [5]. Similar findings have been reported in Australia, Belgium, and Brazil, although whether these changes were caused by vaccine pressure or natural variation has been debated [31–34]. Our data suggest that the rising G2 incidence at the time of vaccine introduction in Malawi was likely due to temporal oscillation as many of the cases occurred in children age-ineligible for vaccination (data not shown) and subsequent G2 detection rates decreased with increasing vaccine coverage.

Despite the apparent lower VE associated with some rotavirus genotypes, this was not associated with an increase of any particular genotypes. Detailed characterization of the outer capsid antigenic regions among G1P[8] strains circulating before and after vaccine introduction will be useful to evaluate any potential vaccine-induced selection of specific antigenic profiles. In addition, in light of the recent emergence of double-reassortant G1P[8] on a DS-1–like genetic backbone [35], whole-genome characterization will be important to assess fully the role of reassortment on vaccine performance against a variety of homotypic and heterotypic strains.

Our finding of VE in HIV-exposed children and in stunted children is important for regions with high prevalence of these conditions, and confirms the immunogenicity findings of recent studies [4, 13]. Lower VE among stunted children may be biologically plausible [10, 36] but despite the differing point estimates, the distinction was not statistically significant [37]. We were unable to estimate the impact of severe acute malnutrition on VE because of absence of premorbid weight in our children. We did not collect discharge weights as surrogate of premorbid weight because children were often discharged once tolerating oral intake with lessening diarrhea even if not fully rehydrated.

Interestingly, we did not find an association between VE and disease severity. This may reflect a referral bias, in that children observed at our hospital were either inpatients, or children undergoing a period of observation prior to discharge. Children with milder disease who were rapidly dismissed were more likely to have been missed by study staff and less likely to produce a fecal sample.

There were some limitations to our study. Maintaining consistent ascertainment efforts over a period approaching 2 decades is challenging. As a result, we were unable to report population-based incidence rates, but have been able to report on rotavirus positivity in stool. Despite 3 years of postvaccine surveillance, analysis of specific strata still suffers from low numbers and wide confidence bounds, precluding adequate power to detect specific VE in risk groups. As vaccine coverage approaches a high baseline, unvaccinated children may no longer be representative of the general child population [38–40]. Residual unvaccinated children may differ in other important ways that increase their risk of disease independent of their lack of vaccine.

## CONCLUSIONS

The rotavirus vaccination program in Malawi has led to persistent reductions in the burden of disease in infants, but has not had apparent impact in older children in whom VE is lower. The increasing age of rotavirus cases behoves ongoing assessment in case waning immunity leads to rebound of disease. Despite differences in VE by genotype, no specific genotypes persistently dominate to suggest vaccine escape. VE is unaffected by HIV exposure and we found no significant difference by stunting. Our findings that rotavirus vaccination provides reliable reductions in disease burden in Malawi are encouraging for other high-burden settings with ubiquitous comorbidity.
